# Fabrication of Colorimetric Textile Sensor Based on Rhodamine Dye for Acidic Gas Detection

**DOI:** 10.3390/polym12020431

**Published:** 2020-02-12

**Authors:** Young Ki Park, Byeong M. Oh, A Ra Jo, Ji Hyeon Han, Jee Young Lim, Hyun Ju Oh, Seung Ju Lim, Jong H. Kim, Woo Sung Lee

**Affiliations:** 1 Smart Textiles R&D Group, Korea Institute of Industrial Technology (KITECH), Ansan 15588, Korea; pyk200087@gmail.com (Y.K.P.); gkscl95@naver.com (J.H.H.); specialg@kitech.re.kr (J.Y.L.); 2 Department of Molecular Science and Technology, Ajou University, Suwon 16499, Korea; hanmir980@ajou.ac.kr; 3 Department of Fiber System Engineering, Dankook University, Yongin 16890, Korea; aravv@naver.com; 4 Technical Textiles R&D Group, Korea Institute of Industrial Technology (KITECH), Ansan 15588, Korea; hjoh33@kitech.re.kr

**Keywords:** rhodamine dye, textile sensor, gas detection, dyeing, printing, pH sensitive

## Abstract

For the immediate detection of gaseous strong acids, it is advantageous to employ colorimetric textile sensors based on halochromic dyes. Thus, a rhodamine dye with superior pH sensitivity and high thermal stability was synthesized and incorporated in nylon 6 and polyester fabrics to fabricate textile sensors through dyeing and printing methods. The spectral properties and solubility of the dye were examined; sensitivity to acidic gas as well as durability and reversibility of the fabricated textile sensors were investigated. Both dyed and printed sensors exhibited a high reaction rate and distinctive color change under the acidic condition owing to the high pH sensitivity of the dye. In addition, both sensors have outstanding durability and reversibility after washing and drying.

## 1. Introduction

As chemical industries have grown gradually, strong acids such as hydrochloric acid, sulfuric acid, and hydrofluoric acid are commonly used in almost all industries worldwide. However, in spite of their usefulness, these strong acids can be dangerous as their accidental ingestion, inhalation, or skin contact may cause intoxication. In particular, gaseous strong acids need more precautions because in case of leakage, they diffuse very quickly without being noticed. In order to detect such leakage at an early stage, many industrial sites have utilized semiconductor-based gas sensors. However, these sensors are expensive and electricity-consuming and require that the worker always carry heavy and bulky equipment. Thus, there is a need for quick and easy detection of gas leakage.

Recently, one of the most promising candidates is a colorimetric sensor made of textiles and halochromic dyes. It detects gases by color change as function of pH, thereby showing a visible signal easily without use of power sources such as electricity. In addition, the colorimetric sensor can be fabricated through a simple and low-cost manufacturing process. Furthermore, compared to conventional sensors, colorimetric sensors offer advantages of high flexibility and applicability on large surfaces.

Until now, colorimetric textile sensors based on halochromic dyes have been widely studied. For example, Van der Schueren et al. fabricated sensors using various halochromic dyes with cotton and nylon fibers by the conventional dyeing method [1]. Carolin Peter et al. used a printing method with ethyl cellulose and bromocresol green to develop a sensor [2]. Chaoqun Zhang et al. and Anshika Agarwal et al. prepared electrospun nanofiber sensors by impregnating halochromic dyes to the polymers such as polyacrylonitrile (PAN) and nylon [3,4]. The sensors fabricated in the previous studies were mainly evaluated for their detection performance against liquid acids or bases. In general, for the detection of gaseous acids or bases, textile sensors must have higher sensitivity than those for liquid detection because the gas concentration per unit area is lower than the liquid one. Thereby, it is required to develop halochromic materials with superior sensitivity for gas detection. Also, most of the halochromic dyes are water-soluble; thus, they can be dropped out after washing of fabricated textile sensors. Therefore, it is advantageous to develop halochromic dyes barely soluble in water but soluble in organic solvents for their incorporation during sensor fabrication.

Herein, a rhodamine derivative with high pH sensitivity and durability was selected as a gas-detecting colorant for textile sensors. Rhodamine dyes are halochromic dyes belonging to the xanthene family; they exhibit sensitive and rapid color change in an acidic environment. Because of their excellent stability and photophysical properties, rhodamine dyes are used in various fields, such as laser [5,6,7], imaging in living cells [8,9,10], fluorescence standards [11], and chemosensors [12,13,14,15].

The fabrication methods of textile sensors using halochromic dyes include dyeing, printing, and electrospinning. The dyeing method consists of dissolving dyes in a solvent, soaking the fiber, and allowing the dyes and fiber to undergo mechanisms such as physical adsorption, mechanical retention, or covalent bond formation [16]. Printing is a method of fixing a printing paste made of a dye and a binder on the textile surface. In the electrospinning method, nano-sized fibers can be obtained by spinning a polymer solution under the influence of applied electric fields. Among these methods, textile sensors fabricated through electrospinning have high reactivity to gases, but poor durability and productivity. On the other hand, the dyeing and printing methods are the cheapest and easiest for dye immobilization as well as facilitate excellent durability.

In this study, a novel rhodamine dye (RhYK) with improved thermal properties and pH sensitivity was synthesized, and textile sensors were fabricated by dyeing and printing methods. In addition, sensitivity, durability, and reversibility of the fabricated textile sensors were investigated.

## 2. Materials and Methods

### 2.1. Materials and Instrumentation

Polyamide adjacent fabric (ISO 105-F03) and polyester adjacent fabric (ISO 105-F04) were obtained from Testfabrics, Inc. (West Pittston, PA, USA) All reagents, solvents, and binders were purchased from Alfa Aesar (Ward Hill, MA, USA) and were used without further purification.

The reactions were monitored by Thin layer chromatography (TLC). Flash column chromatography was carried out using silica gel (200–300 mesh). Elemental analysis (EA) was performed by Flash2000 (Thermo Fisher Scientific, Waltham, MA, USA). Proton Nuclear Magnetic Resonance (^1^H NMR) was recorded on a Jeol JNM-LA400 spectrometer (Jeol, Tokyo, Japan) in Heptadeutero-N,N-Dimethylformamide (DMF-d_7_). Mass spectra were recorded with a Q-TOF 5600 mass spectrometer (AB Sciex, Farmingham, MA, USA). Fourier-transform infrared spectroscopy (FT-IR) and Ultraviolet-visible spectroscopy (UV/Vis) spectra were recorded on Spectrum Two (Perkin Elmer, Waltham, MA, USA) and Shimadzu U-2600 (Shimadzu, Kyoto, Japan), respectively. Thermogravimetric analysis (TGA) was performed using the Q-5000 IR equipment (TA Instruments Inc., New Castle, DE, USA) under a high-purity nitrogen atmosphere. The samples were heated from room temperature to 600 °C at a constant heating rate of 10 °C /min.

### 2.2. Dye Synthesis

2-Hydroxycarbazole (0.01 mol), zinc chloride (0.01 mol), and phthalic anhydride (0.015 mol) were heated at 160 °C for 24 h under a solventless condition. After the reaction mixture was cooled to room temperature, DMF (20 mL) was added to the mixture and stirred for 30 min. The dissolved mixture was added dropwise to water (200 mL (10w% NaCl, 3w% HCl) while stirring. The resulting purple precipitate was obtained by filtration. The crude product was purified by silica gel column chromatography with the acetonitrile/water (containing 0.1% formic acid) gradient system (99:1–50:50). Yield = 74.8%

Elemental analysis (EA): anal, calcd. C32H19N2O3+: C; 74.64, H; 3.90, N; 5.44, O; 9.32, found C;74.17, H; 3.93, N; 5.31, O; 11.54; 1H-NMR (500 MHz, DMF-d7, TMS): δ7.110 (t, 2H), 7.377 (t, 2H), 7.510 (d, 1H), 7.543 (d, 2H), 7.562 (s, 1H), 7.805 (s, 2H), 7.868 (t, 1H), 7.896 (t, 1H), 8.067 (d, 2H), 8.173 (d, 1H), 11.725 (s, 1H); Mass spectroscopy (MS) [M]+ calcd. for C32H19N2O3+ 479.14, found 479.139; FT-IR (cm^−1^): 1718 (C=O), 1615–1457 (C-H), 3400–3200 (O-H); decomposition temperature: 220 °C.

### 2.3. Geometry Optimization of the Dye

Density functional theory (DFT) calculations were carried out with the GAUSSIAN09 package (Gaussian, Wallingford, CT, USA). We used the 6e311þþG(d,p) Pople basis set for all elements and the conventional B3LYP exchange correlation function. The intermolecular interactions were analyzed by examining the core twist angles and the size of substituents. The dihedral angle of the RhYK main body was calculated by measuring the distortion angles of the xanthene and benzoic acid groups.

### 2.4. Solubility Test

The solubility of the synthesized dye in various solvents was examined to determine the maximum dye concentration. The prepared dye was added to the solvents at various concentrations, and the solutions were sonicated for 5 min using an ultrasonic cleaner ME6500E (Mettler Toledo, Columbus, OH, USA). The solutions were left to stand for 48 h 25 °C and checked for precipitation to determine the solubility of the dye.

### 2.5. Fabrication of Optical Textile Sensor

#### 2.5.1. Dyeing

The dyeing of the polyamide fabric was carried out by using the RhYK dye in an IR dyeing machine (Daelim Starlet Co., Ltd., Siheung-si, Korea) at a liquor-to-goods ratio of 50:1 to achieve solvent dyeing. The dyebath was prepared with dye (5% on the weight of fiber (o.w.f.)) and solvent (DMF) without the use of dyeing assistants. Dyeing was commenced at 25 °C. The dyebath temperature was increased at a rate of 3 °C/min to 100 °C, maintained at 100 °C for 60 min, and then rapidly cooled to room temperature. The dyed fabrics were rinsed and dried.

#### 2.5.2. Printing

Screen printing was selected as an alternative method for the fabrication of the textile sensor. Ethyl cellulose (2 g) was dissolved in a mixed solution of ethanol and DMF (10 g each). After complete dissolution, the RhYK dye (0.1 g) and plasticizer (triethyl citrate; 3 g) were added.

The polyester fabric was used as a substrate. Printing was conducted using a screen. The mesh size of the used screen was 200 μm. After printing, the sensor was cured at 80 °C for 5 min.

### 2.6. Gas Test of Fabricated Textile Sensor

**Figure 1:**
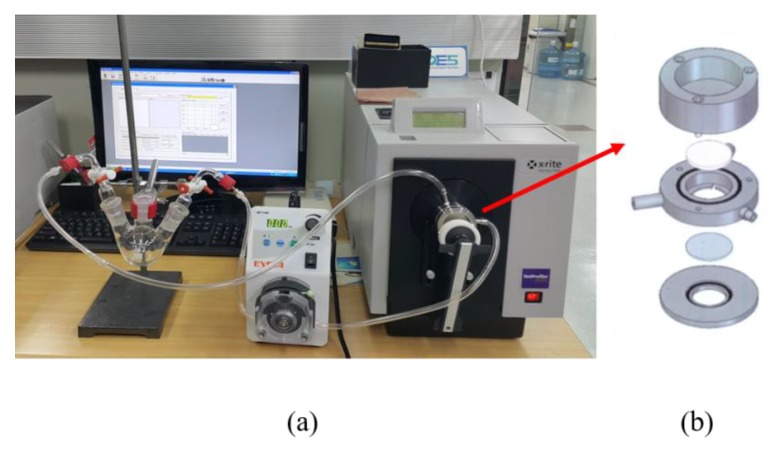
Color measurement system for dynamic color change: (**a**) Entire system; (**b**) Chamber.

A new gas test system was designed, as shown in Figure 1, to measure the color change of the fabricated textile sensors in real time while being exposed to acidic gas. The sensors were placed in a stainless-steel chamber and the acidic gas at a controlled concentration was circulated through the Teflon tube connected to the chamber. Because the internal volume of the entire system was measured, the concentration of the acidic gas could be controlled by the amount of acid injected into the three-neck round bottom flask. The injected acid in the flask was evaporated by a heat gun, and the acidic gas was circulated in the entire system using a peristaltic pump. After exposure to the acidic gas, the dynamic color change of textile sensors was measured by a color-eye 7000A spectrophotometer (X-rite, Grand Rapids, MI, USA). The color change (Δ*E*) and surface color strength (K/S) was calculated using following Equation (1) [17].

(1) $$∆E=\;\sqrt{{\left(∆L^{*}\right)}^{2}+{\left(∆a^{*}\right)}^{2}+{\left(∆b^{*}\right)}^{2}}$$

where L* = lightness, a* = red/green, b* = yellow/blue.

Hydrochloric acid as an acidic gas was used at a concentration of 1 to 100 ppm, and the color change was measured every 10 s for 2 min.

### 2.7. Water-Repellent Finishing and Washing Test

The fabricated sensors were immersed in a 10% solution of a silicone water-repellent agent and squeezed to achieve 100% wet pick-up. After that, dyed and printed sensors were cured at 160 °C and 170 °C for 3 min, respectively. The finished and untreated textile sensors were washed according to the ISO 105 C10—A standard and then subjected to the gas test using 10 ppm of HCl.

### 2.8. Reversibility of Textile Sensors

The fabricated sensors were subjected to the gas test (10 ppm HCl) followed by washing with water (2 min) and drying (50 °C, 5 min) four times in order to calculate their recycle stability.

## 3. Results and Discussion

### 3.1. Synthesis of a Novel Rhodamine Dye (RhYK)

**Scheme 1:**
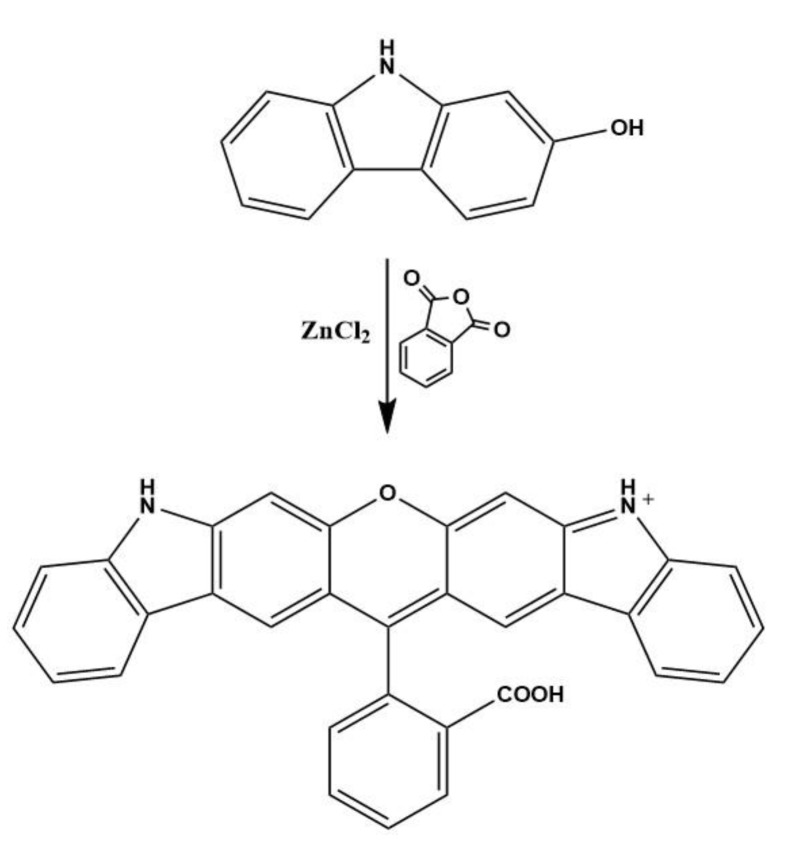
Synthetic route for a novel rhodamine dye (RhYK).

**Figure 2:**
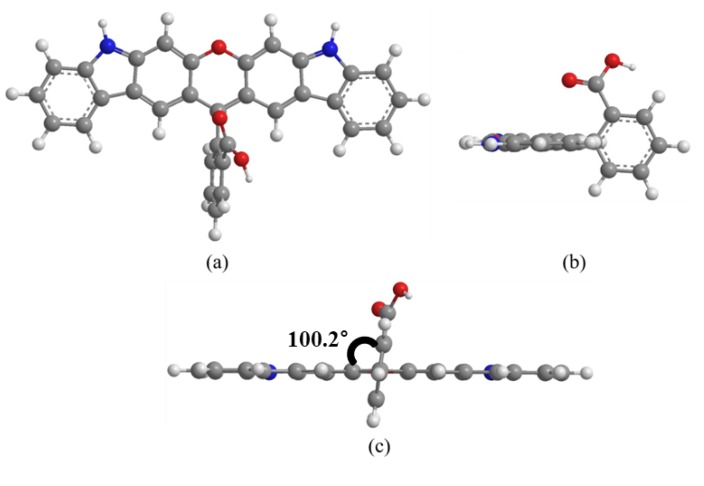
Geometry-optimized structure of the synthesized dye: (**a**) Front; (**b**) Side; (**c**) Top.

The RhYK dye was designed and synthesized on the basis of carbazole to improve its thermal properties. As shown in Scheme 1, a rhodamine derivative was synthesized by two sequential Friedel–Crafts-type reactions of 2-hydroxycarbazole and phthalic anhydride with zinc chloride as the catalyst [18]. The synthesized dye was purified by silica gel column chromatography and characterized by EA, NMR, MS, and FTIR. To explain the structural property of the dye, a geometrically optimized structure was calculated using DFT. Figure 2 shows the optimized structure of the RhYK dye and its dihedral angle. As shown in Figure 2, the RhYK has a rigid and planar structure, which results in a strong π-π interaction. Thus, thermal properties were improved compared to Rhodamine B (T_d_: 210) [19]. In addition, the benzoic acid group allowed for a large dihedral angle of around 100° with the xanthene moiety. The large dihedral angle caused steric hindrances among dye molecules, improving the solubility of the dye in organic solvents.

### 3.2. Spectroscopic Properties of RhYK Dye

Table 1 shows the spectral properties and solubility of the synthesized dye in various solvents. The dye exhibits an absorption maxima (*λ*_max_) in the prepared solvents between 537 and 543 nm, showing a reddish violet color in the solution. The bathochromic shift was observed in the order of increasing polarity of the solvents, which is called positive solvatochromism. This solvatochromic effect is induced by the intramolecular charge transfer (ICT). The first excited state molecule is better stabilized than that of the ground state, with increasing solvent polarity, causing positive solvatochromism [20]. That is, the Highest occupied molecular orbital – Lowest unoccupied molecular orbital (HOMO-LUMO) energy gap is reduced by the dipolar interaction between the dye and the polar solvent.

As shown in Table 1, the molar extinction coefficient at the absorption maxima (*ε*_max_) decreased from 4000 to 500 as the polarity of the solvent increased, indicating a hypochromic effect. The colorless spirolactone form of the RhYK is predominant in the polar environment. Moreover, the charge separation of the dye causes the spirolactoneform to be more polar than the ring-opened form and the hydrogen-bond donor solvent stabilizes the spirolactone form [21]. Hence, the higher polarity of the solvent makes the spirolactone form predominant, resulting in lower *ε*_max_.

**Figure 3:**
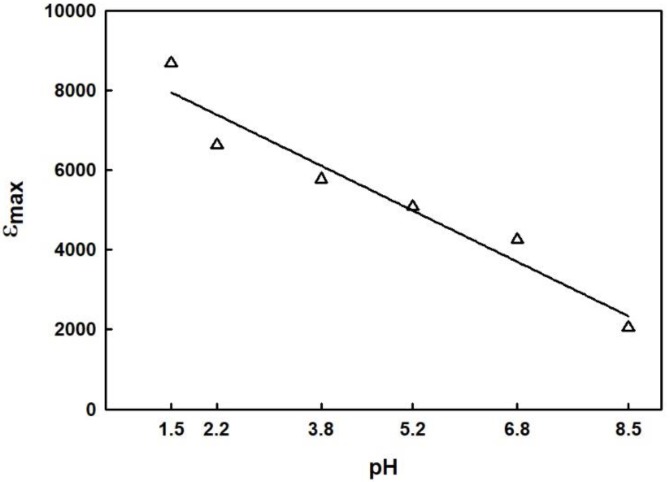
Plot of molar extinction coefficient at 550 nm as a function of pH for RhYK dye (2.61 × 10^−4^ mol·L^−1^) in MeOH.

**Scheme 2:**
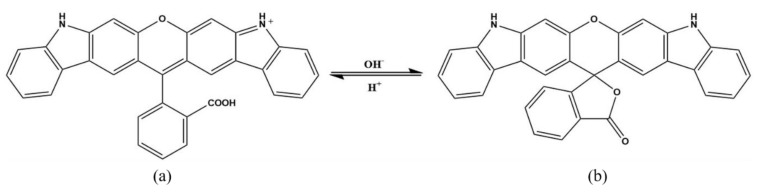
Halochromism of synthesized dye: (**a**) Ring-opened form (violet-red); (**b**) Spirolactone form (colorless).

The change in *ε*_max_ according to varying pH is shown in Figure 3 and Figure S1. The ε_max_ of the dye decreased gradually as the pH of the solution increased. As shown in Scheme 2, this inverse relationship between *ε*_max_ and pH can be explained by the halochromic property of the dye. Under basic conditions, the RhYK dye is converted to the lactone form and the color changes from violet-red to colorless, causing a decrease in *ε*_max_. This large difference in *ε*_max_ enhances visibility after exposure to the acidic gas.

### 3.3. Gas Test of Fabricated Sensor

In this study, the RhYK dye was incorporated into the textiles through dyeing and printing methods. Because the RhYK dye is hydrophobic, it is difficult to employ a conventional dyeing method with water. Instead, the solvent dyeing method was used to dissolve the dye and facilitate dye permeation into the fiber. In case of the printing method, the dye was applied to the textile surface through the screen-printing method in order to fabricate the textile sensor. The gas test of the fabricated sensors was conducted by the method described in Section 2.6.

#### 3.3.1. Dyed Sensor

**Figure 4:**
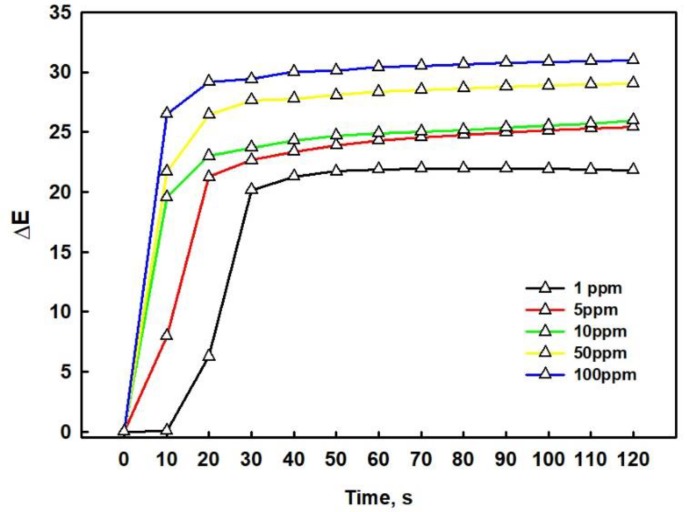
Time-dependent color differences of dyed sensor for gaseous HCl at concentrations from 1 to 100 ppm.

Figure 4 and Figure S2 show the time-dependent color differences of the dyed sensor in response to gaseous HCl at concentrations from 1 to 100 ppm. Table 2 shows the color data of the dyed sensor after exposure to various concentrations of gaseous HCl. Generally, when the color difference (Δ*E*) value is 5 or higher, an observer recognizes two distinctive colors [17]. Therefore, the time taken for the color difference value to reach 5 was measured to evaluate the reaction rate. In addition, the saturated color change (Δ*E_max_*) was measured to evaluate the reactivity. At all concentrations, the reaction rate of the dyed sensor was very high, mostly under 10 s; even at 1 ppm, the reaction occurred within 20 s. Moreover, the Δ*E_max_* value showed a high Δ*E*_max_ value of 21 or more at all concentrations. As the gas concentration increased, the slope of the initial Δ*E* increased sharply, indicating that the reaction rate increased. In addition, Δ*E_max_* increased with the gas concentration, indicating that reactivity and visibility were improved. This is because as the gas concentration increases, the permeability into the fiber and the resulting contact between the gas and the dye increase. Also, as shown in Figure S6 and S8, the fabricated textile sensor using RhYK dye showed superior sensing property and visibility compared to commercial halochromic dye (Bromothymol blue) and representative rhodamine dye (Rhodamine B). In Table S2, the effect of Volatile organic compounds (VOCs) on sensor’s detection performance (poison effect) was confirmed. Since the RhYK dye was designed and synthesized to detect acid gases, they did not react with non-acidic VOCs gases. However, in case of formaldehyde, the color of the sensor changed slightly after immersion owing to weak acidity of formaldehyde. The result indicated the synthesized dye can be suitably applied to textiles for acidic gas detection via common dyeing method.

#### 3.3.2. Printed Sensor

**Figure 5:**
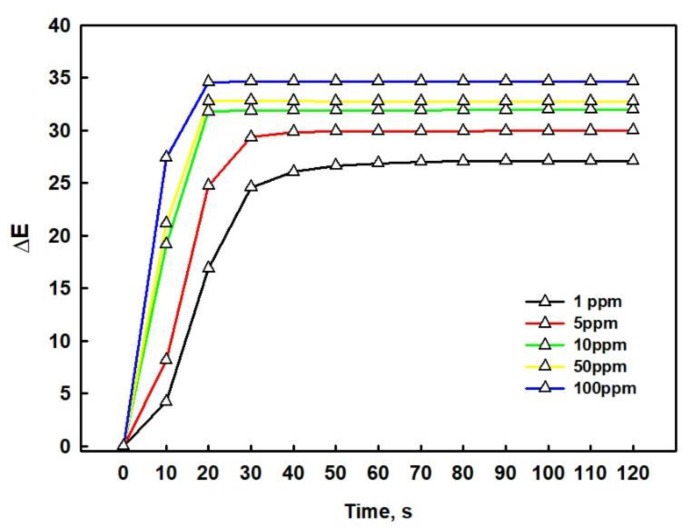
Time-dependent color differences of printed sensor for gaseous HCl at concentrations from 1 to 100 ppm.

Figure 5 and Figure S3 shows the time-dependent color differences of the printed sensor for various concentrations of gaseous HCl (1–100 ppm). Table 3 shows the color data of the dyed sensor after exposure to various concentrations of gaseous HCl. The printed sensors showed a high reaction rate and excellent reactivity at all concentrations; it showed a reaction rate of less than 10 s and a Δ*E* of 27 even at 1 ppm. The reactivity and reaction rate of the printed sensor were better than those of the dyed sensor; these improved properties can be attributed to the fabrication method of the sensors. In the dyeing method, the dye is applied to the surface and interior of the fiber; in contrast, in the printing method, the dye is applied only to the surface. Therefore, the printed sensor has better contact with gas, which results in a higher reaction rate and higher reactivity. Figure S7 shows the sensing property of the printed textile sensors with RhYK dye, Bromothymol blue and Rhodamine B, respectively. Among them, the sensor using Bromothymol blue showed superior sensing property compared to the others. However, the sensor using Bromothymol blue is not suitable to be used as commercial product since it is highly soluble, like many other commercial halochromic dyes, and thus easily removed when washed.

### 3.4. Durability of Fabricated Sensors

The fabricated sensors were finished by a water-repellent agent to minimize the unintended color change of the sensors by an acidic or alkaline liquid. In addition, a washing test was conducted to evaluate sensor durability.

#### 3.4.1. Dyed Sensor

Table 4 shows the color change data according to washing and finishing of the dyed sensor. When the Δ*E*_max_ and saturated K/S values of the untreated and finished samples were compared, those of the finished samples were higher, whether washed or not. This is because the silicone water-repellent agent on the fiber surface lowers the surface refractive index, resulting in an increase in the K/S values [22]. Meanwhile, the initial K/S value barely changes because the initial color of the sensor is almost colorless and not affected by the finishing agent as shown in Table 4 and Figure S4. Hence, the contrast between initial and saturated K/S values, as well as the Δ*E*_max_ values, increased in the finished samples.

Moreover, the Δ*E*_max_ and saturated K/S values of the washed samples slightly decreased than those of the not-washed samples. This is because a small amount of the dye can drop out during the washing test. However, when the Δ*E*_max_ value of the finished and finished & washed samples was compared, the difference between the values of the two samples was much higher than that of the untreated and washed samples. This is because the finishing agent can drop out in addition to the dropped-out dye. When the finishing agent drops out, it does not affect the initial color but reduces the K/S applied to the final color. Thus, it leads to a larger Δ*E*_max_ difference between the finished and finished & washed samples. Despite the drop out of the dye and finishing agent, the fabricated sensors maintain their detection performance after washing because the decrease in the reaction rate and reactivity after washing is small.

#### 3.4.2. Printed Sensor

Table 5 shows the color change data according to washing and finishing of the printed sensor.

When the Δ*E*_max_ and saturated K/S values of the untreated and finished samples were compared, the values of the finished sample were higher because of the change in the refractive index, as described in Section 3.4.1. However, unlike the dyed sensors, the initial K/S value increased because the initial color of the printed sensor is not colorless and affected by the finishing agent as shown in Figure S5.

The decrease in the Δ*E*_max_ and K/S values after washing of untreated samples can be also explained by the dropping out of the dye, as described above. However, in case of the finished sample, the Δ*E*_max_ value increased after washing. This might be because the decrease rate of the initial K/S value between finished and finished &washed sample (0.15–0.09; 40%) is larger than that of the saturated K/S value (1.61–1.31; 18.6%). It means the color strength effect applied to the initial color can be more reduced than that of the final color, thereby increasing the Δ*E*_max_ value after washing.

### 3.5. Reversibility of Fabricated Sensors

The reversibility was measured to evaluate the reusability of the fabricated textile sensors. A cycle of acidic gas detection–washing–drying was performed four times, as described in Section 2.8.

#### 3.5.1. Dyed Sensor

**Figure 6:**
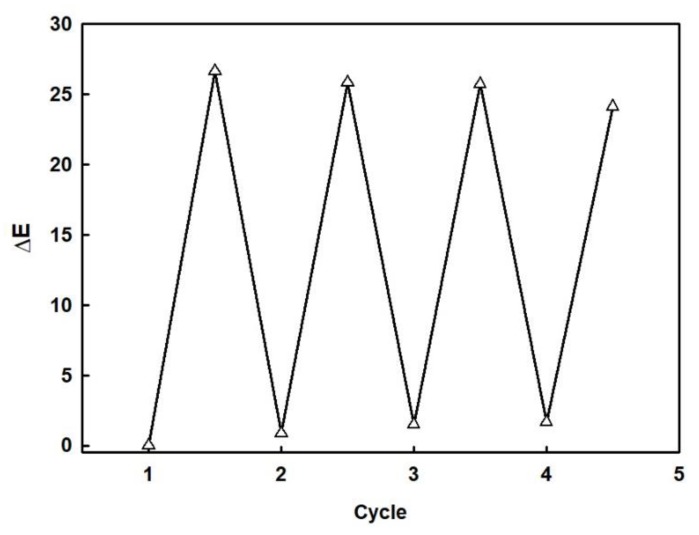
Acidic gas detection cycle of dyed textile sensor.

As shown in Figure 6, as the number of cycle increased, the initial color difference increased and the final color difference decreased. It is considered that this occurs due to the drop out of the dye during washing. Even after washing and drying four times, the color difference before and after detection showed high values over 20, indicating sufficient reusability of the dyed textile sensors.

#### 3.5.2. Printed Sensor

**Figure 7:**
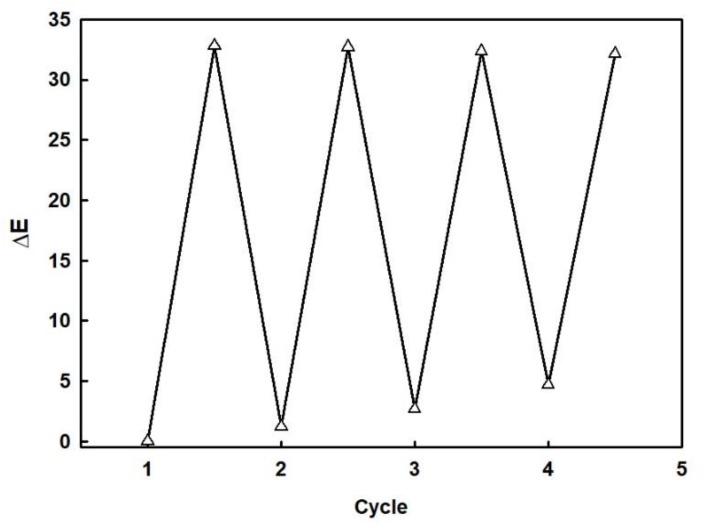
Acidic gas detection cycle of printed textile sensor.

As shown in Figure 7, the printed sensor showed similar results to those of the dyed sensor. The initial color difference value was larger than that of the dyed sensor because the binder on the surface of the printed sensor interfered with the reversible reaction of the dye. Meanwhile, the decrease in the final color difference value is smaller than that of the dyed sensor, because the binder prevents the dye from dropping out during washing. Similar to the dyed sensor, the reusability of the printed sensor can be confirmed by the fact that the final color difference value is above 30 even after washing and drying four times.

## 4. Conclusions

In this study, a novel rhodamine dye with high pH sensitivity and durability was synthesized, and textile sensors based on the dye were fabricated through dyeing and printing methods in order to immobilize the dye on nylon 6 or Polyethylene terephthalate (PET) textiles. Both dyed and printed textile sensors exhibited high reaction rates of less than 10 s and high reactivity even at low concentrations of HCl gas because of the high pH sensitivity of the dye. In addition, saturated color change values of all the sensors were higher than 20, which afforded excellent visibility. The printed sensors showed better detection performance than that of the dyed sensors because of easy accessibility to the gases. The hydrophobic nature of the dye and compatibility among the dye, solvent and binder polymer prevented the loss of dyes after washing, enhancing the durability of the fabricated textile sensors. Also, the change in the refractive index caused by the applied water repellent enhanced the K/S on the colored parts of the textile sensors. Thus, it increased Δ*E*_max_ of the textile sensors as well as minimized the unintended color change caused by the external acid or alkaline liquids. Furthermore, all the sensors showed excellent reversibility even after several cycles of washing and drying. As shown in Table S1, fabricated prototypes based on the dyed and printed textile sensors also showed excellent detection performance against HCl gas. Consequently, these results indicated that the fabricated textile sensor can be a promising candidate for practical sensor applications.

## Figures and Tables

**Table 1:** Spectral properties and solubility of RhYK dye in various solvents.

Solvents	*λ*_max_ (nm)	*ε*_max_ (M^−1^cm^−1^)	Polarity Index	Solubility
MeOH	537.5	4260.54	5.1	+
EtOH	540.5	4256.71	5.2	+
DMF	542.5	517.24	6.4	+++
DMSO	543	572.80	7.2	+++

* +: slightly soluble, ++: soluble, +++: freely soluble.

**Table 2:** Color data of dyed sensors after exposure to various concentrations of gaseous HCl.

ppm	Before	1	5	10	50	100
Color at ΔE_max_	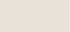	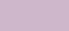	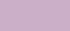	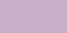	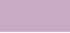	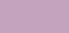
Time at ΔE > 5	-	<20 s	<10 s	<10 s	<10 s	<10 s
ΔE_max_	-	21.96	25.59	25.96	29.14	31.05

**Table 3:** Color data of dyed sensors after exposure to various concentrations of gaseous HCl.

ppm	Before	1	5	10	50	100
Color at ΔE_max_	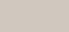	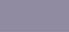	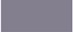	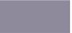	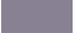	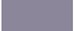
Time at ΔE > 5	-	<10 s	<10 s	<10 s	<10 s	<10 s
ΔE_max_	-	27.10	29.99	30.39	32.74	34.65

**Table 4:** Color change data according to washing and finishing of the dyed sensor.

ppm	Untreated	Washed	Finished	Finished & Washed
Time at Δ*E* > 5	<10 s	<10 s	<10 s	<10 s
Δ*E*_max_	25.96	25.60	29.40	27.68
Initial—Saturated K/S at 550 nm	0.05–0.62	0.04–0.52	0.04–0.69	0.04–0.64

**Table 5:** Color change data according to washing and finishing of the printed sensor.

ppm	Untreated	Washed	Finished	Finished & Washed
Time at Δ*E* > 5	<10 s	<10 s	<10 s	<10 s
Δ*E*_max_	30.39	29.52	30.86	32.32
Initial—Saturated K/S at 550 nm	0.11–1.30	0.09–1.07	0.15–1.61	0.09–1.31

## Supplementary Materials

The following are available online at https://www.mdpi.com/2073-4360/12/2/431/s1, Figure S1: Halochromism of RhYK dye (2.61 × 10^−4^ mol·L^−1^) in MeOH, Figure S2: Time-dependent color change of dyed sensor for gaseous HCl (10 ppm), Figure S3: Time-dependent color change of printed sensor for gaseous HCl (10 ppm), Figure S4: Color strength changes according to washing and finishing of the dyed sensor: before (dashed) and after (solid) gas exposure, Figure S5: Color strength changes according to washing and finishing of the printed sensor: before (dashed) and after (solid) gas exposure, Figure S6: Time-dependent color change of dyed sensors on exposure to gaseous HCl (10 ppm), Figure S7: Time-dependent color change of printed sensors on exposure to gaseous HCl (10 ppm), Figure S8: Color change of fabricated sensors on exposure to gaseous HCl (10 ppm), Table S1: Sensitive color changes of prototype cloths based on dyed and printed textile sensors when exposed to gaseous HCl (100 ppm), Table S2: Effect of VOCs on sensor’s acid gas detection performance (poison effect).
