# Supplementary material for: Fabrication of Colorimetric Textile Sensor Based on Rhodamine Dye for Acidic Gas Detection
Source: Polymers (Basel). 2020 Feb 12;12(2):431. doi: 10.3390/polym12020431 (PMC7077626; doi:10.3390/polym12020431)
Supplement: Supplementary file 1 [file polymers-12-00431-s001.pdf]

Fabrication of colorimetric textile sensor based on Rhodamine dye for acidic gas detection

Young Ki Park, Byeong M. Oh, A Ra Jo, Ji Hyeon Han, Jee Young Lim, Hyun Ju Oh, Seung Ju Lim, Jong H. Kim and Woosung Lee

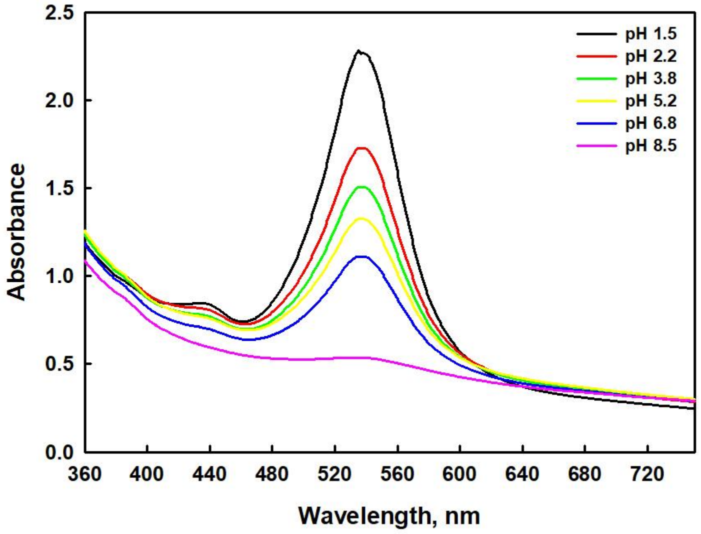

Figure S1. .Halochromic property of RhYK dye (2.61 x 10^{-4} mol·L^{-1}) in MeOH.

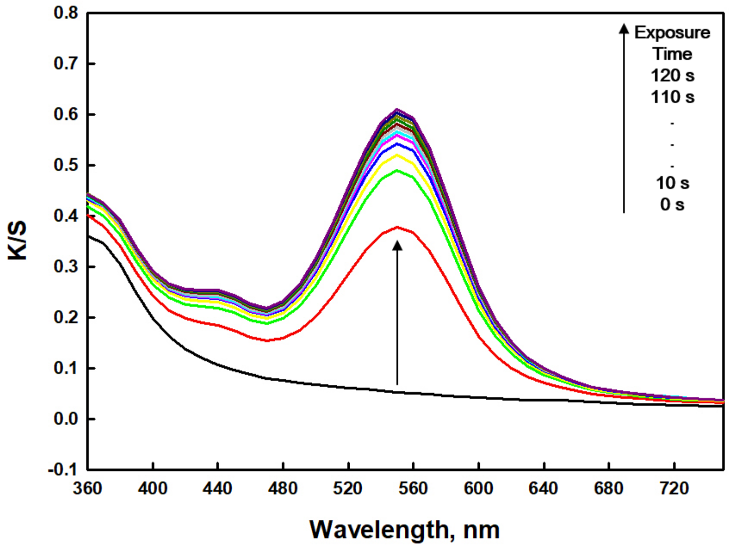

Figure S2. Time-dependent color change of dyed sensor on exposure to gaseous HCl (10 ppm).

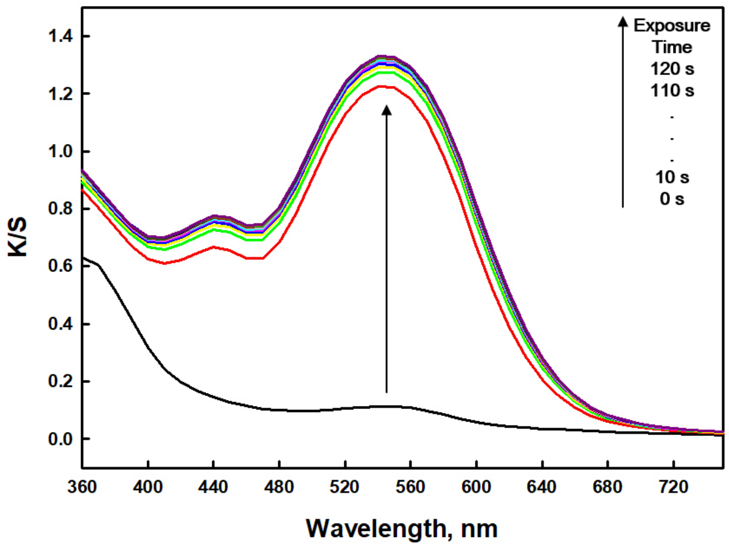

Figure S3. Time-dependent color change of printed sensor on exposure to gaseous HCl (10 ppm).

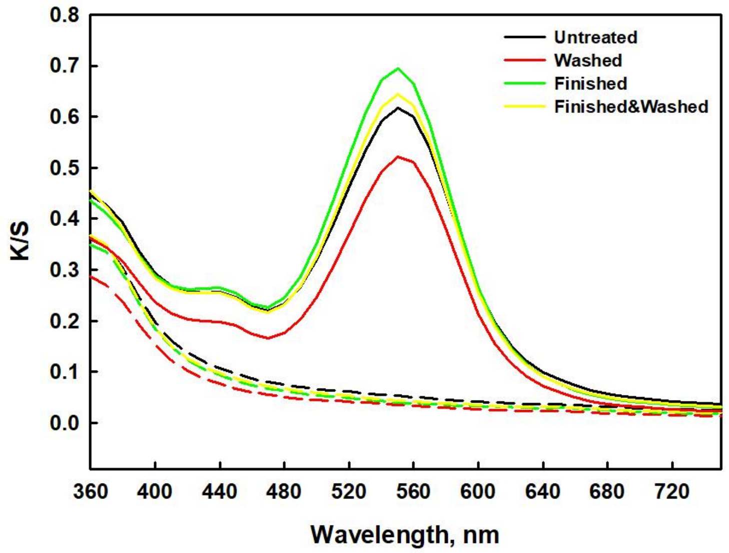

Figure S4. Color strength changes according to washing and finishing of the dyed sensor: before (dashed) and after (solid) gas exposure.

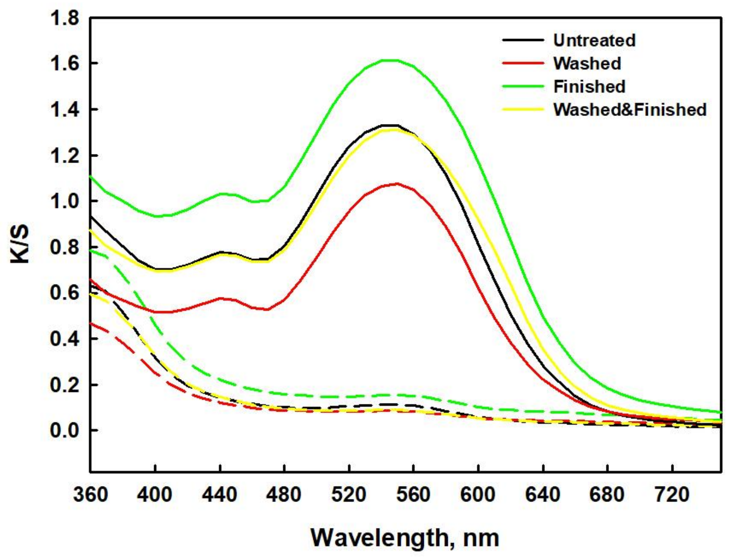

Figure S5. Color strength changes according to washing and finishing of the printed sensor: before (dashed) and after (solid) gas exposure.

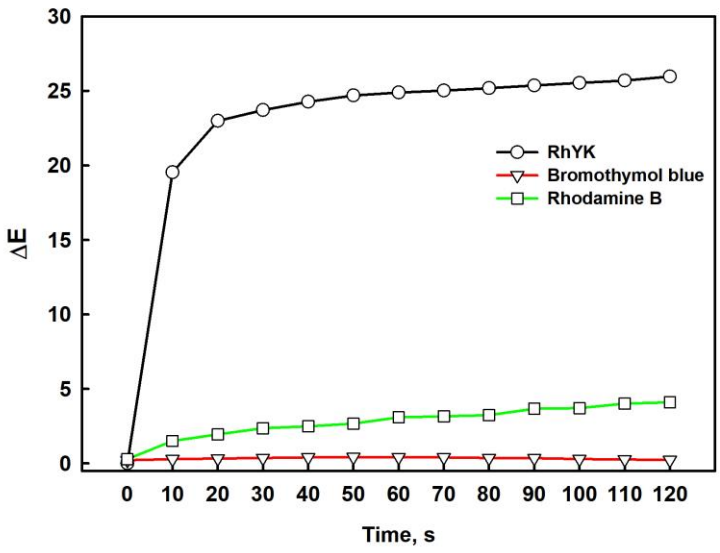

Figure S6. Time-dependent color change of dyed sensors on exposure to gaseous HCl (10 ppm).

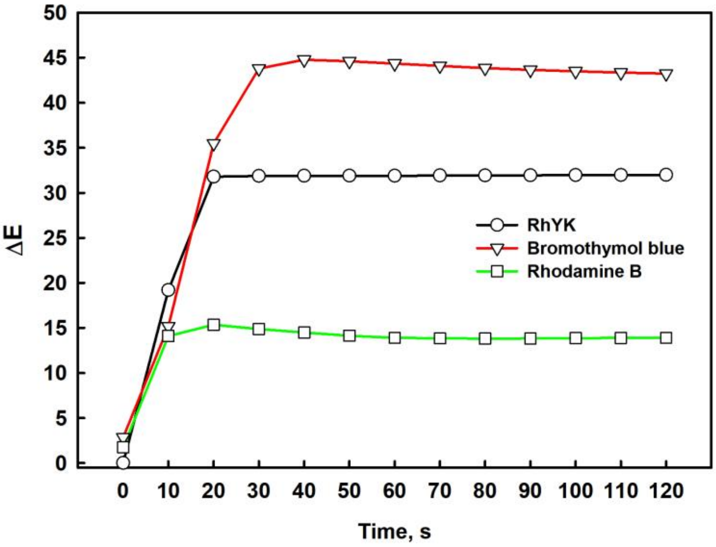

Figure S7. Time-dependent color change of printed sensors on exposure to gaseous HCl (10 ppm).

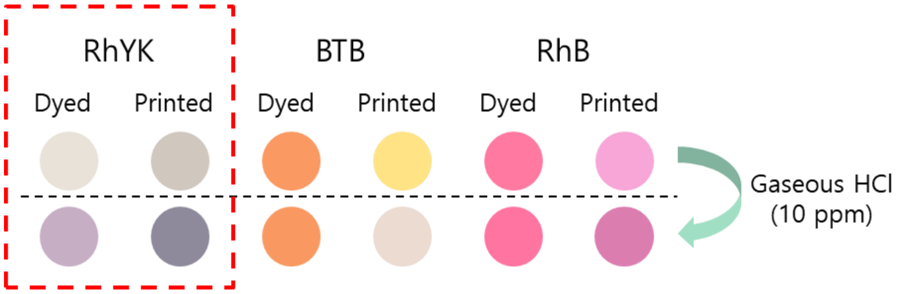

Figure S8. Color change of fabricated sensors on exposure to gaseous HCl (10 ppm).

Table S1. Sensitive color changes of prototype cloths based on dyed and printed textile sensors when exposed to gaseous HCl (200 ppm).

Types of fabrication	Before	After (2 min)
Dyed	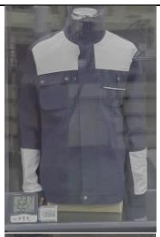	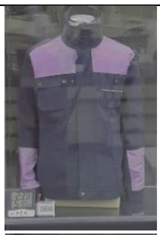
Printed	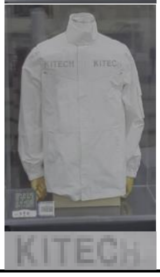	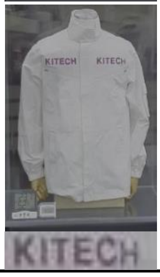

Table S2. Effect of VOCs on sensor's acid gas detection performance (poison effect).

VOCs	Immersion	After Immersion	After Exposure to Gaseous HCl
Methanol	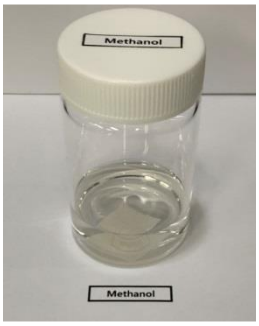	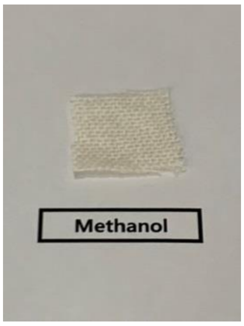	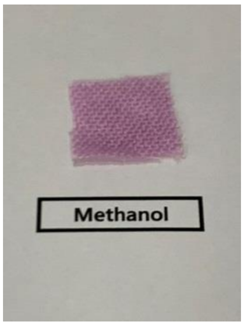
Ethanol	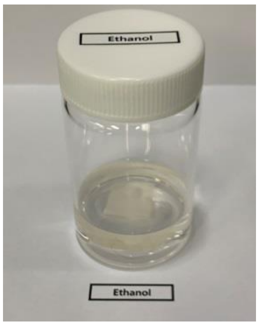	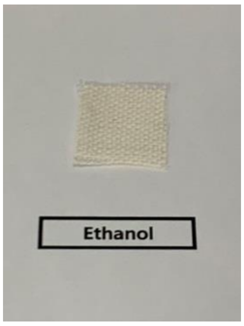	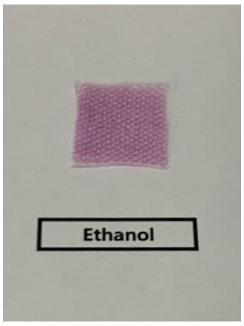
Toluene	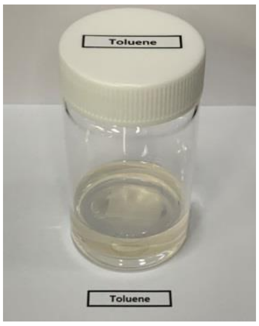	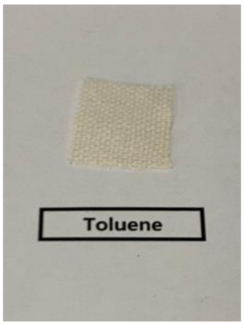	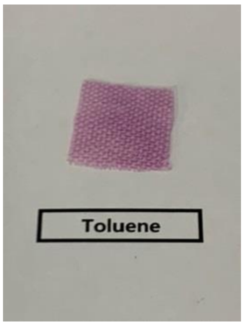
Formaldehyde	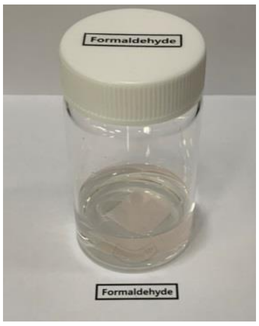	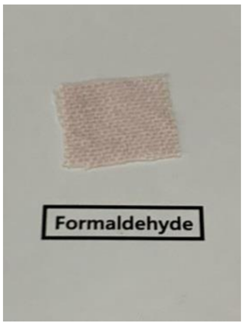	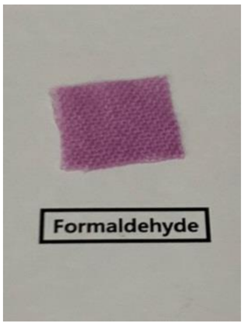
